# A Biotinylated cpFIT-PNA Platform for the Facile Detection of Drug Resistance to Artemisinin in *Plasmodium falciparum*

**DOI:** 10.1021/acssensors.3c02553

**Published:** 2024-03-06

**Authors:** Odelia Tepper, Daniel H. Appella, Hongchao Zheng, Ron Dzikowski, Eylon Yavin

**Affiliations:** † The Institute for Drug Research, The School of Pharmacy, The Faculty of Medicine, The Hebrew University of Jerusalem, Hadassah Ein-Kerem, Jerusalem 9112102, Israel; ‡ Synthetic Bioactive Molecules Section, Laboratory of Bioorganic Chemistry (LBC), National Institute of Diabetes and Digestive and Kidney Diseases (NIDDK), National Institutes of Health, 8 Center Drive, Room 404, Bethesda, Maryland 20892, United States; § Department of Microbiology and Molecular Genetics, The institute for Medical Research Israel - Canada, The Kuvin Center for the Study of Infectious and Tropical Diseases, The Hebrew University-Hadassah Medical School, Jerusalem 9112102, Israel

**Keywords:** FIT-PNA, artemisinin, drug resistance, *P. falciparum*, pfK13

## Abstract

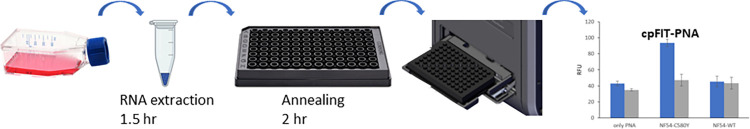

The evolution of drug resistance to many antimalarial drugs in the lethal strain of malaria (*Plasmodium falciparum*) has been a great concern over the past 50 years. Among these drugs, artemisinin has become less effective for treating malaria. Indeed, several *P. falciparum* variants have become resistant to this drug, as elucidated by specific mutations in the pfK13 gene. This study presents the development of a diagnostic kit for the detection of a common point mutation in the pfK13 gene of *P. falciparum*, namely, the C580Y point mutation. FIT-PNAs (forced-intercalation peptide nucleic acid) are DNA mimics that serve as RNA sensors that fluoresce upon hybridization to their complementary RNA. Herein, FIT-PNAs were designed to sense the C580Y single nucleotide polymorphism (SNP) and were conjugated to biotin in order to bind these molecules to streptavidin-coated plates. Initial studies with synthetic RNA were conducted to optimize the sensing system. In addition, cyclopentane-modified PNA monomers (cpPNAs) were introduced to improve FIT-PNA sensing. Lastly, total RNA was isolated from red blood cells infected with *P. falciparum* (WT strain – NF54-WT or mutant strain – NF54-C580Y). Streptavidin plates loaded with either FIT-PNA or cpFIT-PNA were incubated with the total RNA. A significant difference in fluorescence for mutant vs WT total RNA was found only for the cpFIT-PNA probe. In summary, this study paves the way for a simple diagnostic kit for monitoring artemisinin drug resistance that may be easily adapted to malaria endemic regions.

Malaria remains a significant public health concern, particularly in endemic countries. According to the latest World Health Organization (WHO) report from 2022,^1^ an estimated 247 million cases of malaria occurred worldwide in 2021, with 95% of these in Africa. The disease causes over 600,000 deaths annually.

One of the challenges in malaria control and treatment is the development of drug resistance in parasite populations.^2,3^ Almost every drug used to treat malaria has faced the emergence of resistance. Resistance can occur at different stages of the parasite’s life cycle. In some cases, drug resistance is associated with single nucleotide polymorphisms (SNPs) in the parasite’s genome.

For example, in *P. falciparum* parasites, SNPs have been associated with resistance to various antimalarial drugs. Chloroquine resistance is associated with mutations in the Pfcrt gene,^4^ while mefloquine resistance is linked to mutations in the Pfmdr1 gene.^5,6^ Other drugs like sulfadoxine (Pfdhps),^7^ pyrimethamine (Pfdhfr),^8^ and more recently, artemisinin (PfK13)^9,10^ have also shown been associated with specific SNPs that contribute to resistance.

Artemisinin and its derivatives (ARTs) are the recommended first-line drugs for treating malaria, particularly in artemisinin combination therapies (ACTs), where they are used in combination with partner antimalaria drugs.

Artemisinin resistance has primarily been observed in *P. falciparum* parasites in Southeast Asia.^11^ This resistance is associated with multiple nonsynonymous mutations in a specific region of the Kelch protein (K13) in the parasite. Some of the well-known mutations include C580Y, R539T, and Y493H, with C580Y being the dominant mutation (ca. 55%) in resistant *P. falciparum* strains in Southeast Asia.^12^ These mutations in K13 have been linked to a reduced clearance of parasites in ART-treated malaria patients.

Given the rapid emergence of drug resistance, there is a need for new approaches to diagnose drug resistance in a simple, fast, and manageable manner, especially in malaria-endemic regions. While there have been attempts to develop SNP detection methods for point-of-care treatment, the current detection methods mainly rely on PCR-based technologies^13−15^ and isothermal amplification techniques. These methods, such as loop-mediated isothermal amplification (LAMP),^16−19^ drop-digital PCR^20^ and molecular beacon PCR,^21^ single-nucleotide primer extension (SNPE),^22^ and fluorescence resonance energy transfer-melting curve analysis (FRET-MCA),^23^ are highly sensitive but are often time-consuming and may require expensive equipment, sensitive materials (polymerases), and skilled personnel. These requirements pose challenges in resource-limited malaria-endemic countries, where such facilities and expertise may not be readily available.

A simple approach for designing RNA sensing molecules is based on peptide nucleic acids (PNA); a fully synthetic DNA mimic that exhibits high affinity and specificity to complementary DNA/RNA sequences.^24,25^

PNA-based RNA/DNA sensors have been developed by various chemical approaches,^26−36^ among them, FIT-PNAs (forced intercalation-peptide nucleic acids).^37−46^

FIT-PNAs incorporate a cyanine dye (e.g., thiazole orange) in place of a canonical nucleobase of the PNA sequence (a.k.a. surrogate base). The presence of the dye allows for a large increase in fluorescence upon hybridization with the target DNA/RNA, as the dye’s intramolecular rotation is restricted in the duplex form, preventing radiationless decay channels.^47^

FIT-PNAs as well as their RNA/DNA versions (FIT probes) were shown to discriminate RNA sequences at a single base resolution.^48−55^

Cyclopentane-modified PNAs (cpPNAs) are PNA monomers with a cyclopentane backbone that have a defined stereochemistry.^56−60^

cpPNAs have been shown to exert outstanding binding affinity to complementary DNA/RNA with even single substitutions resulting in a dramatic increase in melting temperatures (*T*_m_).^61^ We have recently reported that placing the BisQ fluorophore (a red-emitting surrogate base) between two cyclopentane-modified PNA monomers (cpFIT-PNA) increases the quantum yield (and brightness) by about 2-fold upon hybridization with fully complementary RNA.^62^ In addition, cpFIT-PNAs were shown to improve mismatch discrimination, in particular for the case of pyrimidine–pyrimidine mismatches.

We have previously reported FIT-PNAs as RNA sensing molecules that detect the C580Y SNP in *P. falciparum* infected red blood cells (iRBCs).^54^ In this recent study, FIT-PNAs were incubated with iRBC and analyzed by FACS and confocal microscopy. These analyses, however, limit the use of such RNA sensors in most endemic countries due to the lack of resources and trained technicians for such high-tech equipment.

To overcome this limitation, we report the development of a simple chemical approach for providing cpFIT-PNA RNA sensors for point of care use. The general approach is to label the cpFIT-PNA with a biotin tag to allow its simple attachment to a streptavidin-coated 96-wells plate. In turn, this allows an easy setup for adding total RNA (isolated from *P. falciparum*-infected red blood cells) to cpFIT-PNA coated wells followed by a simple fluorescence readout on a 96-well plate reader.

## Materials and Methods

### General

Manual solid-phase synthesis was performed by using 5 mL polyethylene syringe reactors (Phenomenex) that are equipped with a fritted disk. HPLC purifications and analysis were performed on a Shimadzu LC-1090 system using a semipreparative C18 reversed-phase column (Jupiter C18, 5 μ, 300 Å, 250 × 10 mm, Phenomenex) at 50 °C. Eluents: A (0.1% TFA in water) and B (MeCN) were used in a linear gradient (11–40%B in 30 min) with a flow rate of 4 mL/min.

MS measurements for all PNA molecules were measured on a ThermoQuest Finnigan LCQ-Duo ESI mass spectrometer. RNA oligomers were purchased from IDT Inc. (USA). Dry DMF was purchased from Acros and Fmoc/Bhoc protected PNA monomers from PolyOrg Inc. (USA). Fmoc cpT PNA and Fmoc cpC PNA monomers^59^ as well as BisQ monomer^39^ were prepared according to the literature. Fmoc-protected d-lysine, biotin, and reagents for solid phase synthesis were purchased from Merck (Germany). The PEG linker (Fmoc-8-amino-3,6-dioxaoctanoic) was purchased from ChemScene Inc. and black, streptavidin-coated, 96-well plates, C-bottom, were purchased from Greiner bio-one Inc.

### Solid-Phase Synthesis of Competitor PNAs, Biotin-Labeled FIT-PNAs, and Biotin-Labeled cpFIT-PNAs

Biotin-K13 FIT-PNAs, biotin-cpFIT-PNAs, and competitor PNAs (Tables 1 and 2) were synthesized (Scheme 1) and fully characterized by HPLC and ESI-MS (Figures S1–S16). All PNAs and FIT-PNAs were synthesized on the solid support (Novasyn TGA resin, 0.25 mmol/g) by standard solid phase peptide chemistry using Fmoc-protected PNA, cpPNA, and BisQ monomers. A short PEG linker (FmocNHCH_2_CH_2_OCH_2_CH_2_COOH) was introduced (×3) as a spacer between the biotin label and the FIT-PNA sequence (Scheme 1).

**Table 1:**
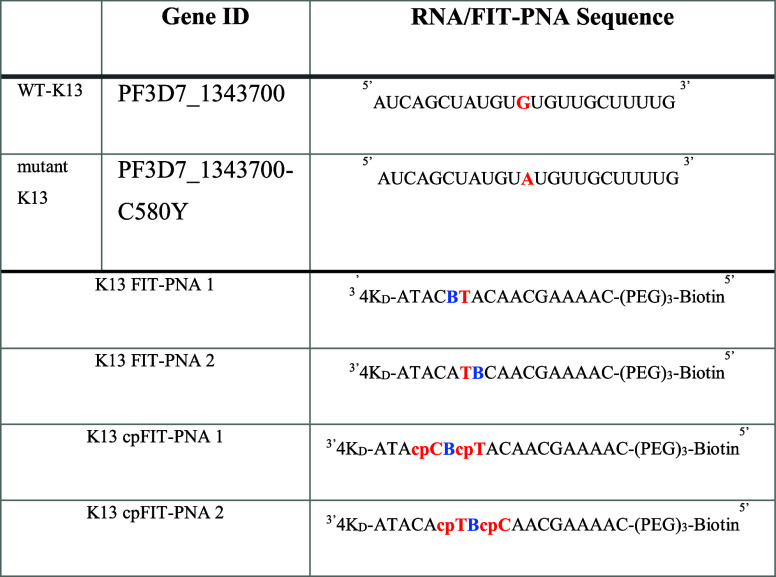
K13 Gene for the WT and Mutant *P. falciparum* as well as FIT-PNA and cpFIT-PNA Sequences Conjugated to Biotin via a Short PEG Linker^a^

a C580Y point mutation in K13 gene is marked in bold red; the BisQ fluorophore is marked in bold blue. Cyclopentane PNA monomers are denoted as “cp” and are marked in bold red. 4KD = 4 d-Lysines.

**Scheme 1:**
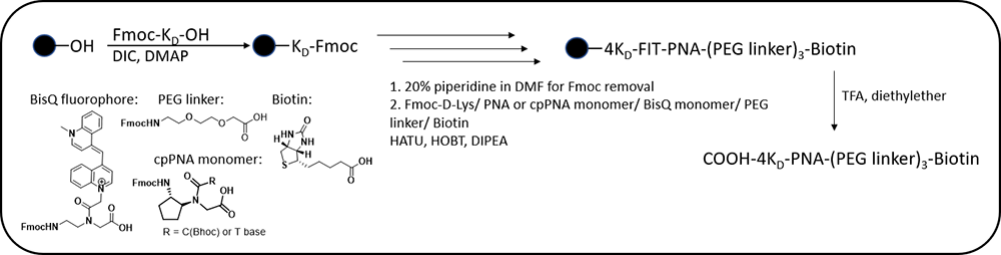
Solid-Phase Synthesis of Biotin-Labeled FIT-PNAs and cpFIT-PNAs

**Table 2:** PNA and DNA Sequences as Competitors That Target WT-K13 mRNA

name	sequence
short competitor 10 mer PNA	^3′^4K_D_-TACA**C**ACAAC^5′^
short competitor 11 mer PNA	^3′^4K_D_-ATACA**C**ACAAC^5′^
short competitor 12 mer PNA	^3′^4K_D_-GATACA**C**ACAAC^5′^
short competitor 13 mer PNA	^3′^4K_D_-CGATACA**C**ACAAC^5′^
full length WT DNA	^3′^ CGATACA**C**ACAACGAAA^5′^

### Fluorescence Measurement with Synthetic RNAs with/without PNA Competitors

Biotin-FIT-PNA (0.5 μM in Tris-Tween buffer (25 mM Tris-HCl, 25 mM EDTA, 150 mM NaCl with 0.05% Tween-20)) was added to a 96-well Greiner streptavidin-coated microplate (C-bottom, black) for 1 h at RT followed by washing (×3) with the same buffer. Next, 0.25 μM synthetic RNA was added for annealing to the biotin-FIT-PNA at 37 °C for 2 h, in the presence/absence of 0.625 μM of the competitor PNA. Finally, wells were washed twice with the Tris-Tween buffer, and the fluorescence was measured on a Cytation 3 plate reader (λex = 587 nm, λem = 619 nm). Limit of detection (LOD) analysis was conducted similarly. After binding of the FIT-PNAs and washing, synthetic C580Y RNA was added at different concentrations. Fluorescence was measured on a Cytation 3 plate reader. Limit of detection was calculated by the equation: LOD = 3.2 × σ/slope.

### Cell Culture and Cell Lysis

All parasite cultures (*P. falciparum*, either WT strain – NF54-PF3D7_1343700-WT or mutant strain – NF54-PF3D7_1343700-C580Y^54^) were cultivated at 5% hematocrit in RPMI 1640 medium, 0.5% Albumax II (Invitrogen), 0.25% sodium bicarbonate, and 0.1 mg/mL gentamicin. Parasites were incubated at 37 °C in an atmosphere of 5% oxygen, 5% carbon dioxide, and 90% nitrogen. The level of parasitemia was calculated by counting 3 independent blood smears stained with Giemsa under a light microscope. To 20 mL of unsynchronized parasite culture, with parasitemia of 3–5%, was added 200 μL of 5% saponin for red blood cell (RBC) lysis. The culture was tilted a few times and centrifuged (4000 rpm, 4 min). The parasites’ pellet was suspended in 750 μL of Trizol for RNA extraction.

### Total RNA Extraction

Total RNA from parasites was extracted using an Invitrogen RNA isolation kit according to the manufacture instructions. Total RNA concentration was measured with a NanoDrop instrument (NanoDrop 2000c Spectrophotometer, Thermo scientific). Total RNA was diluted with Tris-Tween buffer to a concentration of 200 ng/μL.

### Fluorescence Measurement with Total RNA

To biotin-FIT-PNA or biotin-cpFIT-PNA (0.5 μM) preassembled to a 96-well Greiner streptavidin-coated microplate (C-bottom, black), a total of 200 ng/μL of extracted parasite total RNA was added to each well for annealing (90 min at 37 °C), and the fluorescence was recorded on a Cytation 3 plate reader (λex = 587 nm, λem = 619 nm).

## Results and Discussion

Recently,^54^ we have developed a series of FIT-PNA molecules that target the K13 SNP in *P. falciparum* and provided a proof of concept that these RNA sensors discriminate between wild-type and mutant (C580Y SNP) pfK13 mRNA, as corroborated by FACS analysis and fluorescence microscopy.

Based on these findings and with the aim of developing a diagnostic kit for the detection of artemisinin drug resistance in *P. falciparum*, we have selected the FIT-PNA sequence presented in Table 1. K13 FIT-PNA 1 and K13 FIT-PNA 2 have identical sequences except that the location of the surrogate base (BisQ, B in Table 1) is positioned at 3′ (FIT-PNA 1) or 5′ (FIT-PNA 2) to the point mutation (T base shown in red in Table 1 that is complementary to the A base in the mutant K13 mRNA).

To allow the assembly of these RNA sensors to a platform (96-well streptavidin-coated plate), we introduced 3 short PEG linkers between the biotin tag and the FIT-PNA sequence at the 5′-end. In addition, we introduced 4 d-lysines at the 3′-end of the FIT-PNA to render these molecules with water solubility.

Considering the general design of FIT-PNAs, we have found that, in comparison to the (PEG)_3_ linker, using a short linker (one PEG) separating the biotin label from the FIT-PNA resulted in a negligible increase in fluorescence after RNA hybridization for a different mutation in *P. falciparum* (pfCRT gene; data not shown). In addition, the choice of the 17-mer PNA sequence was based on our previous design of C580Y FIT-PNAs.^54^

The general synthetic approach is presented in Scheme 1.

To generate brighter RNA sensors, we have also prepared the cyclopentane PNA analogs K13 cPFIT–PNAs 1 and 2. This design was based on a previous study^62^ that points to the added benefit of introducing cpPNA monomers that flank the BisQ surrogate base as means of increasing the brightness of the RNA sensor (when hybridized to the target complementary RNA) by ca. 2-fold.

**Figure 1:**
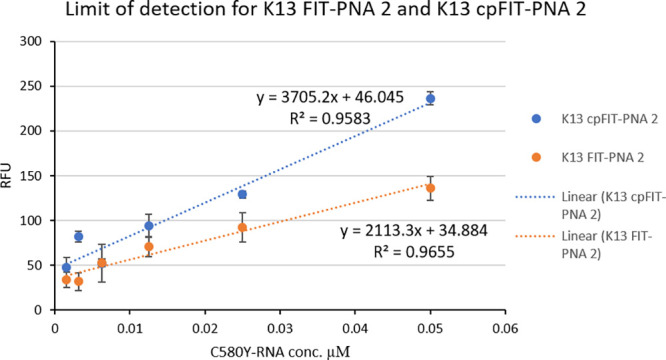
Fluorescence measurements for K13 FIT-PNA 2 and K13 cPFIT–PNA 2 on streptavidin plates for determining LOD. After FIT-PNA binding (0.5 μM, 1 h, RT) and washing, fully matched synthetic C580Y RNA was added at different concentrations and allowed to anneal at 37 °C for 2 h. Fluorescence was measured on a Cytation 3 plate reader (λex = 580 nm, λem = 610 nm, *n* = 3). Limit of detection was calculated by the equation: LOD = 3.2 × σ/slope.

We next compared the limit of detection (LOD) of K13 cPFIT–PNA 2 to that of the nonmodified FIT-PNA (K13 FIT-PNA 2) using synthetic mutant RNA (C580Y). As shown in Figure 1, values of 9 and 16 nM were obtained for both streptavidin-bound FIT-PNAs, respectively. The introduction of cp monomers to K13 FIT-PNA (K13 cPFIT–PNA 2) results in a lower LOD that is consistent with the higher sensitivity of this probe. This value is comparable to the detection of DNA (LOD = 2 nM) on a biotinylated molecular beacon-type sensor attached to a streptavidin surface.^63^

The biotin-labeled FIT-PNAs (K13 FIT-PNA 1 and 2, 0.5 μM) were dissolved in TRIS-buffer containing 0.05% Tween-20 and added to streptavidin-coated wells for a 2 h incubation period at 37 °C to allow complete FIT-PNA attachment. The wells were then washed with buffer (×3) and a solution of 0.625 μM synthetic RNA (see Table 1 for sequences) was added to the wells. Both K13 FIT-PNA sequences showed a small difference between the WT and mutant RNAs (Figure S17). A similar behavior was observed for cyclopentane modified (K13 FIT-cpPNA 1 and K13 FIT-cpPNA 2) as an increase in fluorescence was observed for both WT and mutant RNA sequences (Figure S18).

As an approach to improve the discrimination between mutant and WT RNA targets, 4 PNA competitors were designed that span the wild-type sequence (Table 2). For these PNAs, a short peptide (4 d-lysines) was introduced at the 3′-end of the sequence to improve water solubility. Namely, the shorter PNA competitors would sequester the wild-type RNA sequence, thereby decreasing the signal generated after the addition of WT RNA to the wells. This decrease in signal is anticipated to have a negligible effect on the mutant RNA target, thus providing a simple approach to improve SNP discrimination.

All 4 competitor PNAs (Table 2) were tested for all 4 FIT-PNAs presented in Table 1. The experiments were conducted by adding the PNA competitor (0.65 μM) just prior to the addition of the target RNA (WT or mutant).

In all cases, an improvement in discrimination was observed as detailed in Figure 1 for K13 FIT-PNA 2 and K13 cPFIT–PNA 2 and as summarized in Table 3. All other fluorescence measurements are detailed in the SI (Figures S17 and S18). A full-length DNA competitor (spanning all 17 bases of the WT PNA sequence) was also tested and found to be inferior to competitor PNAs (Figures S17 and S18).

**Table 3:** K13 Discrimination Ratios with/without PNA Competitors, Defined as the Ratio between the Fluorescence of the Duplex with Synthetic Mutant RNA (fdsMut) and the Fluorescence of the Duplex with Synthetic WT RNA (fdsWT) for K13 FIT-PNAs and K13 cPFIT–PNAs

	f_ds_Mut/f_ds_WT	f_ds_Mut/f_ds_WT	f_ds_Mut/f_ds_WT	f_ds_Mut/f_ds_WT
	K13 FIT-PNA 1	K13 **cp**FIT-PNA 1	K13 FIT-PNA 2	K13 **cp**FIT-PNA 2
with RNA only	1.82	1.32	1.85	2.1
10 mer PNA competitor	2.57	2.38	2.35	4.06
11 mer PNA competitor	2.87	2.54	**2.99**	**4.96**
12 mer PNA competitor	2.65	2.74	1.62	3.55
13 mer PNA competitor	2.5	2.49	2.29	3.25
full length WT-DNA	2.13	1.44	2.04	2.82

**Figure 2:**
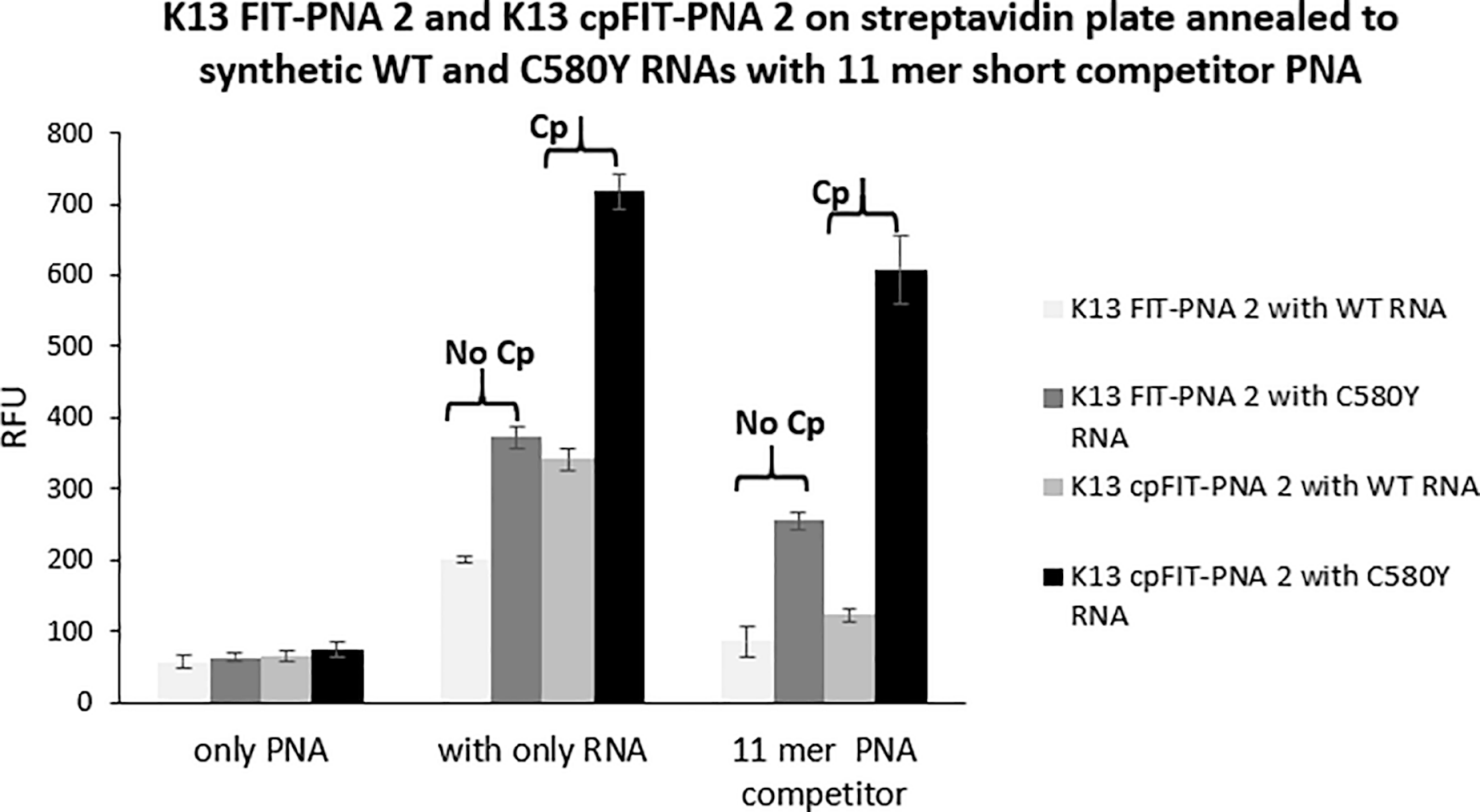
Fluorescence measurements with synthetic RNA. K13 FIT-PNA 2 (0.5 μM) and K13 cPFIT–PNA 2 (0.5 μM) fluorescence after their assembly on a streptavidin plate with the synthetic mutant (C580Y) and WT RNAs (0.625 μM) in the absence or presence of the 11-mer PNA competitor (0.65 μM). Fluorescence was measured on a Cytation 3 plate reader (λex = 587 nm, λem = 619 nm, *n* = 3).

As shown in Figure 2, the addition of the 11-mer competitor PNA has a remarkable effect on the fdsMut/fdsWT ratio, providing a 3-fold and 5-fold difference for K13 FIT-PNA 2 and K13 cPFIT–PNA 2, respectively.

Two strains of *P. falciparum* were grown in a cell culture (red blood cells, RBC): the wild type strain (NF54-WT) and the mutant strain (NF54-C580Y), which harbors the C580Y SNP in the K13 gene. After reaching high parasitemia (over 5%), infected RBC were detached and underwent cell lysis. Thereafter, total RNA from each strain was extracted and isolated.

**Figure 3:**
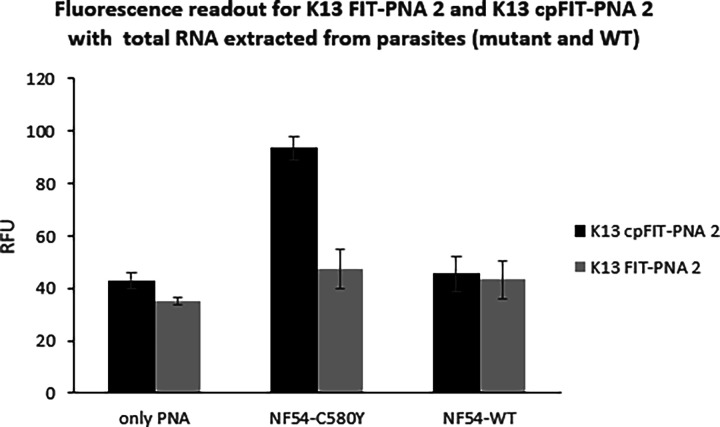
Fluorescence measurements with total RNA extracted from parasites. Fluorescence measurements for K13 FIT-PNA 2 and K13 cPFIT–PNA 2 fluorescence on a streptavidin plate with extracted WT-RNA (NF54-WT) and mutant C580Y RNA (NF54-C580Y). [RNA] = 200 ng/μL per well was added after K13 FIT-PNA 2/K13 cPFIT–PNA 2 binding (0.5 μM, 1 h, RT). Fluorescence was measured on a Cytation 3 plate reader (λex = 587 nm, λem = 619 nm, *n* = 3).

Initial attempts to detect total RNA in the presence of a PNA competitor resulted in a negligible fluorescence readout. However, in the absence of PNA competitor, the discrimination of mutant K13 RNA from WT was highly appreciable, as shown in Figure 3.

**Scheme 2:**
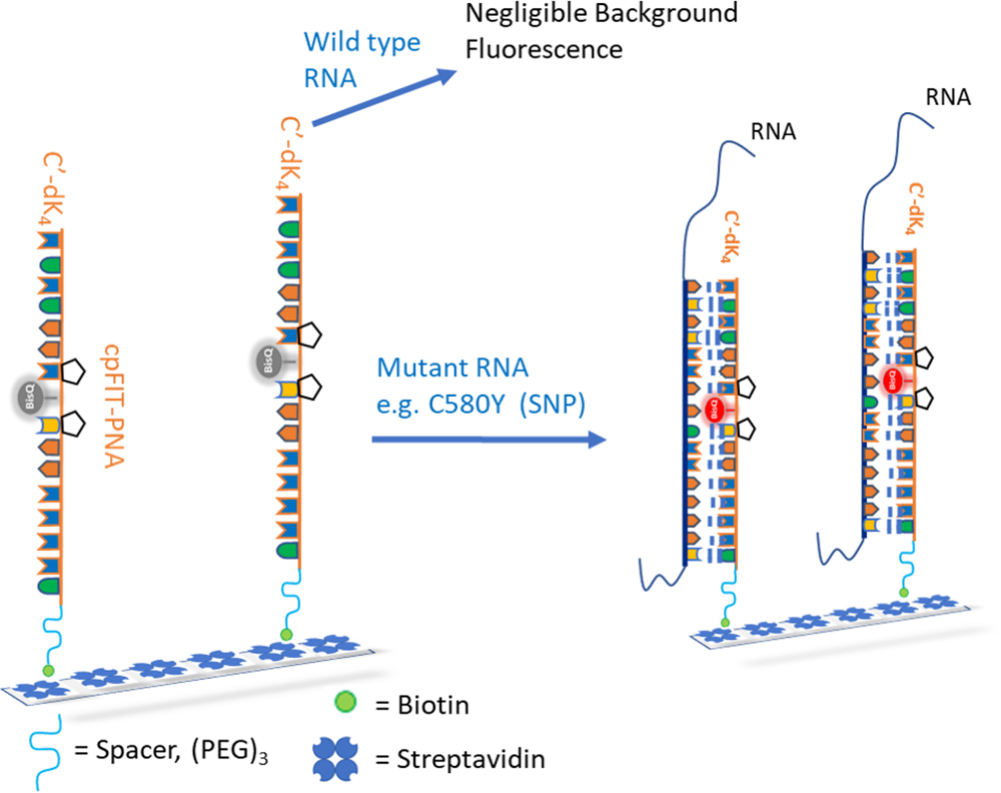
Strategy to Develop a Kit for Discriminating Mutant over Wild-Type RNA by Attaching the Biotin-Labelled cpFIT-PNA to a Streptavidin Surface

Thus, the overall strategy for SNP detection by cpFIT-PNA is presented in Scheme 2.

In recent years, several diagnostic approaches have been devised to allow a relatively simple approach to identify the C580Y SNP in the K13 gene that is associated with *P. falciparum* drug resistance to artemisinin and its derivatives (e.g., artesunate). These include the use of loop-mediated isothermal amplification (LAMP),^16,17,19^ a method based on amplification of the readout signal with DNA primers. In the current study, we show, for the first time, a simple sensing system for detecting this SNP without the need of amplifying the signal. The detection of this type of SNP is challenging as the mismatch for the RNA sensor hybridized to wild-type K13 mRNA results in a G:U mismatch (G:U wobble) that is well tolerated in RNA duplexes.^64^

Indeed, after assembly of biotin K13 FIT-PNAs or biotin K13 cPFIT–PNAs to streptavidin plates, the addition of synthetic RNA (WT or C580Y) did not result in a substantial difference between RNAs (Figure 2 and Figures S17 and S18). This has prompted us to synthesize PNA competitors (Table 2) that were designed to target the WT RNA sequence as means of decreasing the fluorescent signal originating from the WT-RNA:K13 FIT-PNA duplex. This approach was highly rewarding as the ratio between fluorescence of the duplexes with synthetic RNA (fdsMut)/(fdsWT) increased from about 2-fold without PNA competitors to ca. 4-fold and 5-fold for cpFIT-PNA 2 with either 10-mer or 11-mer PNA competitors, respectively (Table 3).

This strategy did not seem to add any value once the sensing system was tested with total RNA extracted from the parasites (data not shown). On the contrary, the addition of the PNA competitor (e.g., 11-mer) hampered the readout fluorescent signal on the plate reader. One possible explanation for this observation may be related to binding of the PNA competitor to RNA at different sites that are not the targeted site (WT K13 mRNA). This is reasonable given the short sequence (10 to 13 mers) that have many binding sites in the genomic RNA molecule.

Nonetheless, assembly of the cyclopentane-modified K13 FIT-PNA (Biotin K13 cPFIT–PNA 2, Table 1) followed by the addition of total RNA extracted from the 2 different *P. falciparum* strains (Figure 3) resulted in a ca. 2-fold difference between mutant and WT total RNAs ((fdsMut)/(fdsWT)). The data also point to the added value of introducing cyclopentane PNA monomers that flank the BisQ surrogate. Biotin K13 FIT-PNA 2 lacking the cyclopentane PNA monomers did not produce a significant difference between the C580Y and WT total RNAs. Given the simplicity of this sensing system (i.e., assembly of the RNA sensor on the plate and addition of extracted RNA), we envision the use of this sensing system in malaria endemic regions that are limited in resources and in well-trained medical lab technicians.

## Conclusions

This study reports the development of a simple RNA sensing platform that is designed to detect a single point mutation (SNP) that is associated with drug resistance to the flag antimalarial drug, artemisinin. Biotin-labeled cpFIT-PNAs were assembled onto streptavidin plates. Simply adding total RNA extracted from the mutant strain of *P. falciparum* (NF54-C580Y) resulted in a substantial increase in the fluorescent readout in comparison to the fluorescent signal obtained with the wild type strain (NF54-WT). This sets the ground to introduce this sensing system to malaria endemic regions (e.g., Myanmar, Thailand, and Cambodia) where artemisinin drug resistance is emerging.

## Supporting Information Available

HPLC and ESI-MS of FIT-PNAs, cpFIT-PNA and competitor PNA, fluorescence measurements of all FIT-PNAs and cpFIT-PNA with synthetic RNA in the presence/absence of PNA or DNA competitors (PDF)
