# Supplementary material for: A Biotinylated cpFIT-PNA Platform for the Facile Detection of Drug Resistance to Artemisinin in Plasmodium falciparum
Source: ACS Sens. 2024 Mar 6;9(3):1458–64. doi: 10.1021/acssensors.3c02553 (PMC10964236; doi:10.1021/acssensors.3c02553)
Supplement: Supplementary file 1 — se3c02553_si_001.pdf [file se3c02553_si_001.pdf]

Supporting Information

# A biotinylated cpFIT-PNA platform for the facile detection of drug resistance to Artemisinin in *Plasmodium falciparum*

Odelia Tepper[a], Daniel H. Appella[b], Hongchao Zheng[b], Ron Dzikowski[c], and Eylon Yavin[*a]

[a]The Institute for Drug Research, The School of Pharmacy, The Faculty of Medicine, The Hebrew University of Jerusalem, Hadassah Ein-Kerem, Jerusalem 9112102, Israel.

[b]Synthetic Bioactive Molecules Section, Laboratory of Bioorganic Chemistry (LBC), National Institute of Diabetes and Digestive and Kidney Diseases (NIDDK), National Institutes of Health, 8 Center Drive, Room 404, Bethesda, Maryland 20892, United States.

[c]Department of Microbiology and Molecular Genetics, The institute for Medical Research Israel - Canada, The Kuvin Center for the Study of Infectious and Tropical Diseases, The Hebrew University-Hadassah Medical School, Jerusalem 9112102, Israel.

* To whom correspondence should be addressed. Tel: +972-2-6758692; Fax: +972-2-6757574; Email: eylony@ekmd.huji.ac.il.

Supporting Information

**Tables of contents:**


General procedures and materials 3

HPLC and ESI-MS of FIT-PNA and cpFIT-PNAs 4-7

HPLC and ESI-MS of short competitor PNAs 8-11

Fluorescence readouts for K13 FIT-PNA 1 and K13 cpFIT-PNA 1 with all 4 competitor PNAs and DNA 12

Fluorescence readouts for K13 FIT-PNA 2 and K13 cpFIT-PNA 2 with all 4 competitor PNAs and DNA 13

References 14

## General procedures and materials

Manual solid-phase synthesis was performed by using 5 mL polyethylene syringe reactors (Phenomenex) that are equipped with a fritted disk. HPLC purifications and analysis were performed on a Shimadzu LC-1090 system using a semi-preparative C18 reversed-phase column (Jupiter C18, 5u, 300Å, 250x10mm, Phenomenex) at 50ºC. Eluents: A (0.1% TFA in water) and B (MeCN) were used in a linear gradient with a flow rate of 4mL/min. Mass analysis of FIT-PNAs was acquired on a TSQ Quantum Access Max (Thermo Fisher Scientific, Basel, Switzerland) mass spectrometer. The analysis was performed by direct injection into the mass spectrometer using electrospray ionization (ESI) in positive mode and full scan analysis (range of 200–1500 m/z).

RNA oligos were purchased from IDT, USA. Fmoc/Bhoc protected PNA monomers were purchased from PolyOrg Inc. (USA). Fmoc-D-Lysine and reagents for solid phase synthesis were purchased from Merck (Germany) and Biolab (Israel). Fmoc-protected cyclopentane PNA monomers (C and T) and BisQ were synthesized as previously reported [1, 2].

Solid phase synthesis of cpFIT-PNA and FIT-PNA/PNAs was done as in [3] and as described in manuscript.

**Fluorescence spectrometry** measurements were conducted as reported in [3].

Supporting Information

<u>HPLC and MS of FIT-PNAs and cpFIT-PNAs:</u>

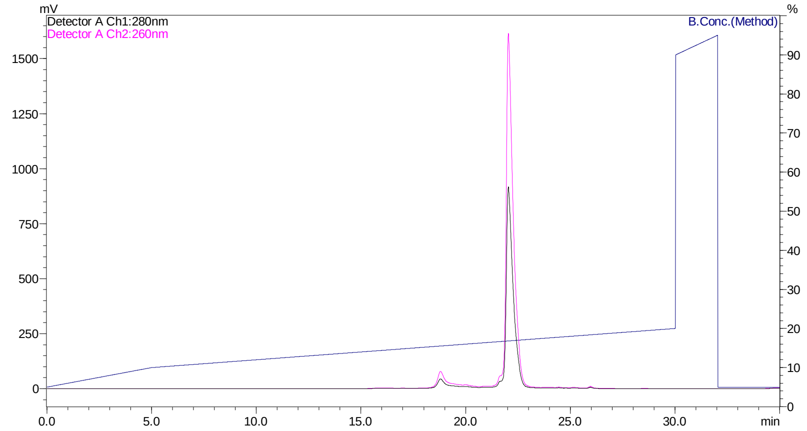


**Fig. S1:** HPLC chromatogram of K13 FIT-PNA 1. Eluents: A (0.1% TFA in water) and B (MeCN) were used in a linear gradient (10-15 % B in 30min) with a flow rate of 4mL/min.

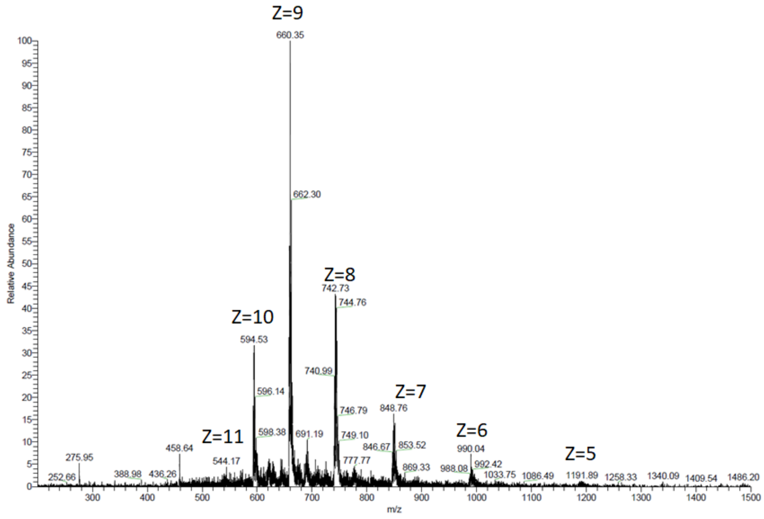


**Fig. S2:** ESI-MS of K13 FIT-PNA 1. $M_{calc}$ =5936.65, $M_{obs}$ =5934.4.

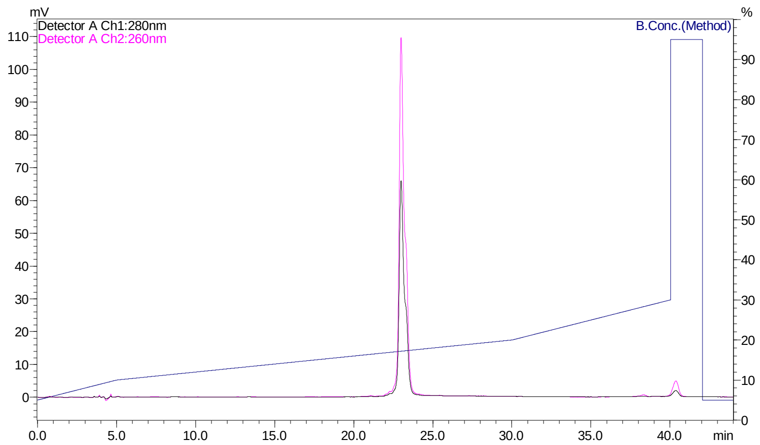


**Fig. S3:** HPLC chromatogram of K13 cpFIT-PNA 1. Eluents: A (0.1% TFA in water) and B (MeCN) were used in a linear gradient (10-15 % B in 30min) with a flow rate of 4mL/min.

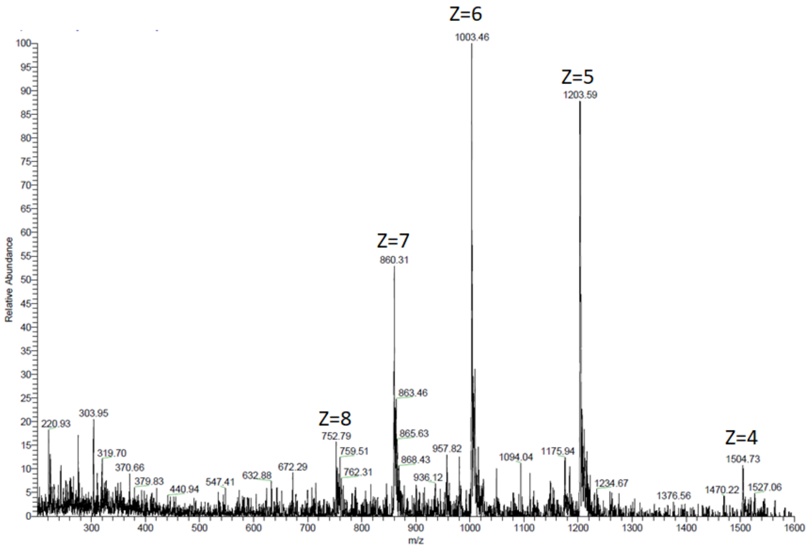


**Fig. S4:** ESI-MS of K13 cpFIT-PNA 1. $M_{calc}$ = 6016.72, $M_{obs}$ =6014.3.

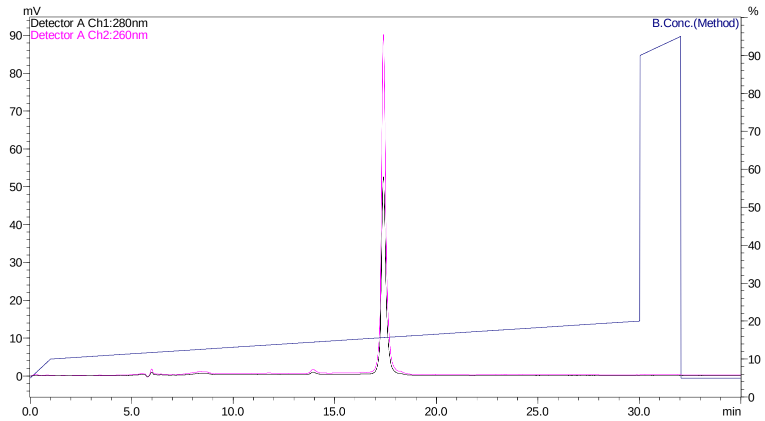


**Fig. S5:** HPLC chromatogram of K13 FIT-PNA 2. Eluents: A (0.1% TFA in water) and B (MeCN) were used in a linear gradient (10-15 % B in 30min) with a flow rate of 4mL/min.

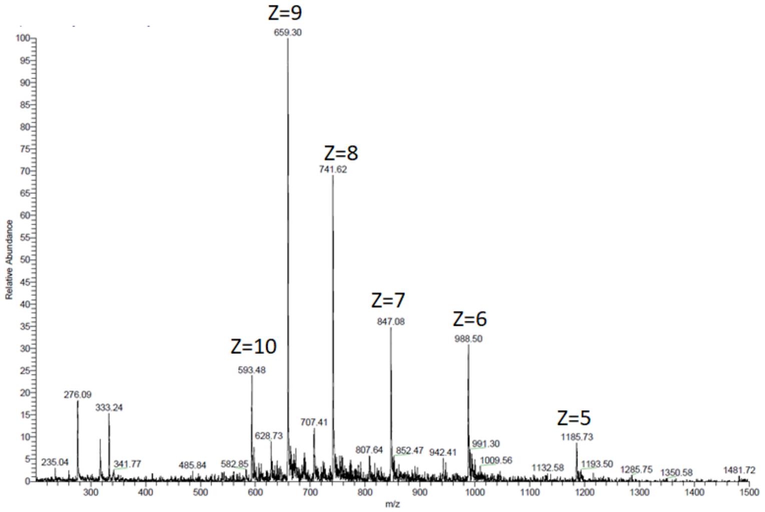


**Fig. S6:** ESI-MS of K13 FIT-PNA 2. $M_{calc}$ =5922.64, $M_{obs}$ =5924.3.

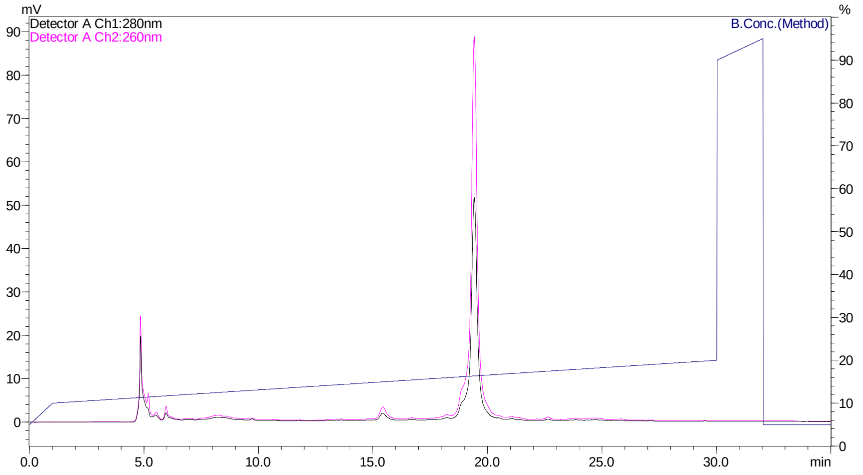


**Fig. S7:** HPLC chromatogram of K13 cpFIT-PNA 2. Eluents: A (0.1% TFA in water) and B (MeCN) were used in a linear gradient (10-15 % B in 30min) with a flow rate of 4mL/min.

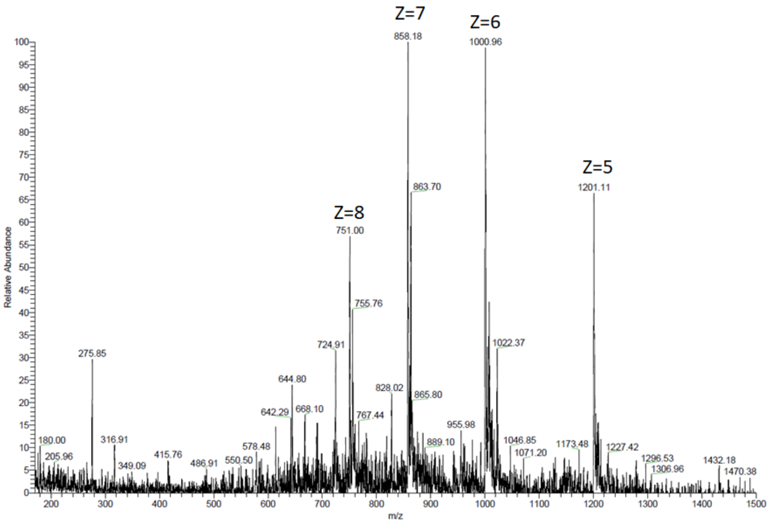


**Fig. S8:** ESI-MS of K13 cpFIT-PNA 2. $M_{calc}$ = 6002.7, $M_{obs}$ =6000.14.

HPLC and MS of short competitor PNAs:

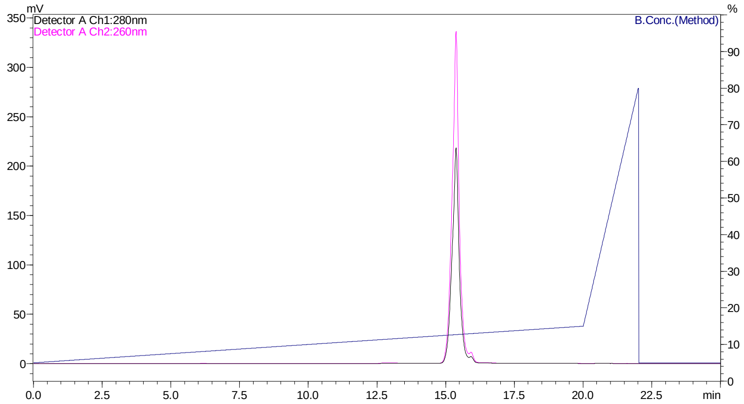


**Fig. S9:** HPLC chromatogram of short competitor 10 mer PNA. Eluents: A (0.1% TFA in water) and B (MeCN) were used in a linear gradient (10-15 % B in 30min) with a flow rate of 4mL/min.

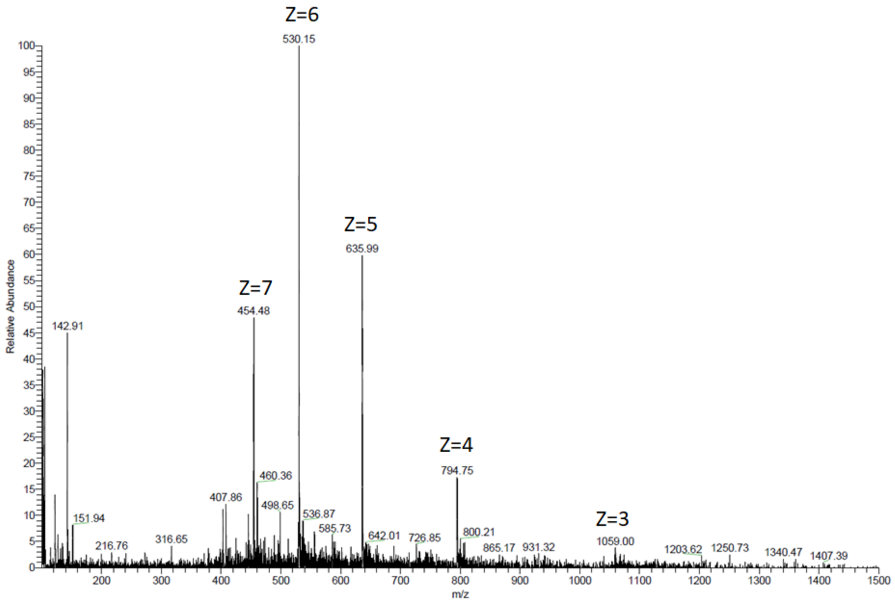


**Fig. S10:** ESI-MS of short competitor 10 mer PNA. $M_{calc}$ =3176.46, $M_{obs}$ =3174.8.

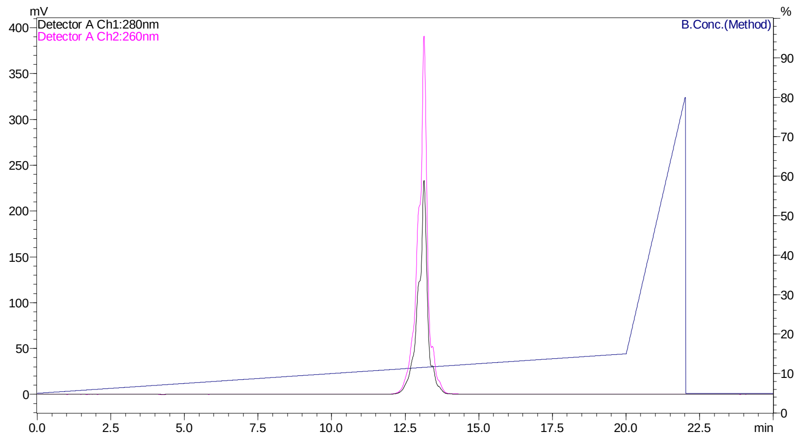


**Fig. S11:** HPLC chromatogram of short competitor 11 mer PNA. Eluents: A (0.1% TFA in water) and B (MeCN) were used in a linear gradient (10-15 % B in 30min) with a flow rate of 4mL/min.

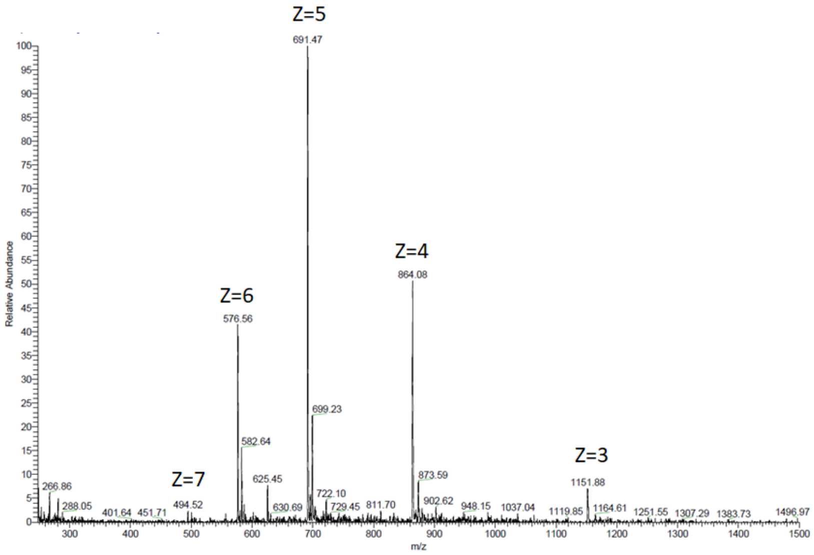


**Fig. S12:** ESI-MS of short competitor 11 mer PNA. $M_{calc}$ =3451.58, $M_{obs}$ =3452.4.

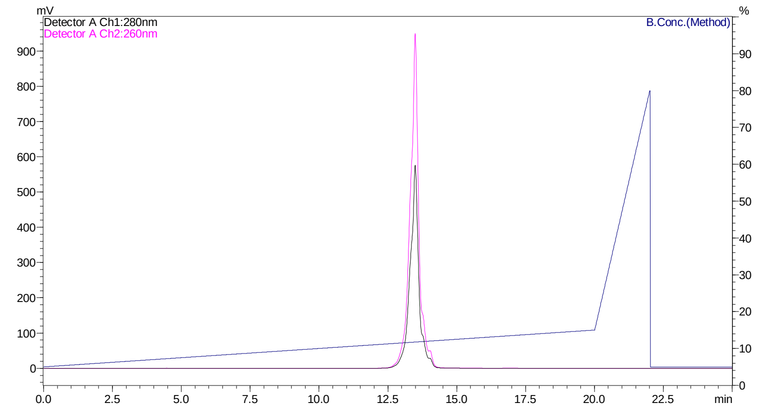


**Fig. S13:** HPLC chromatogram of short competitor 12 mer PNA. Eluents: A (0.1% TFA in water) and B (MeCN) were used in a linear gradient (10-15 % B in 30min) with a flow rate of 4mL/min.

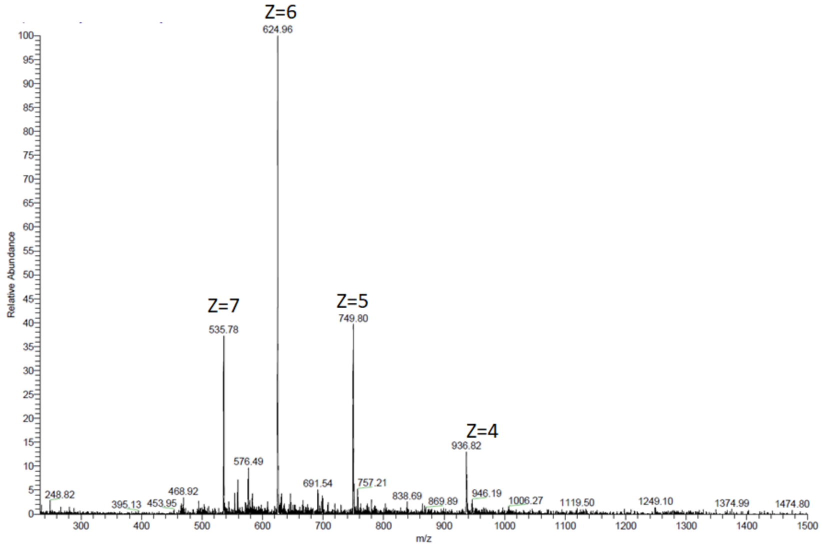


**Fig. S14:** ESI-MS of short competitor 12 mer PNA. $M_{calc}$ =3742.69, $M_{obs}$ =3743.6.

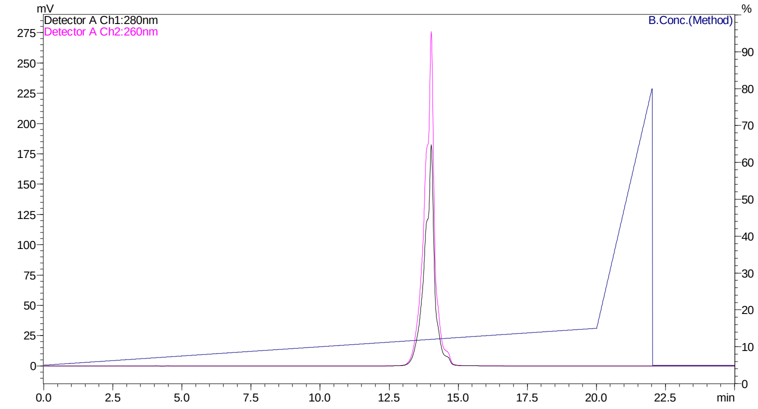


**Fig. S15:** HPLC chromatogram of short competitor 13 mer PNA. Eluents: A (0.1% TFA in water) and B (MeCN) were used in a linear gradient (10-15 % B in 30min) with a flow rate of 4mL/min.

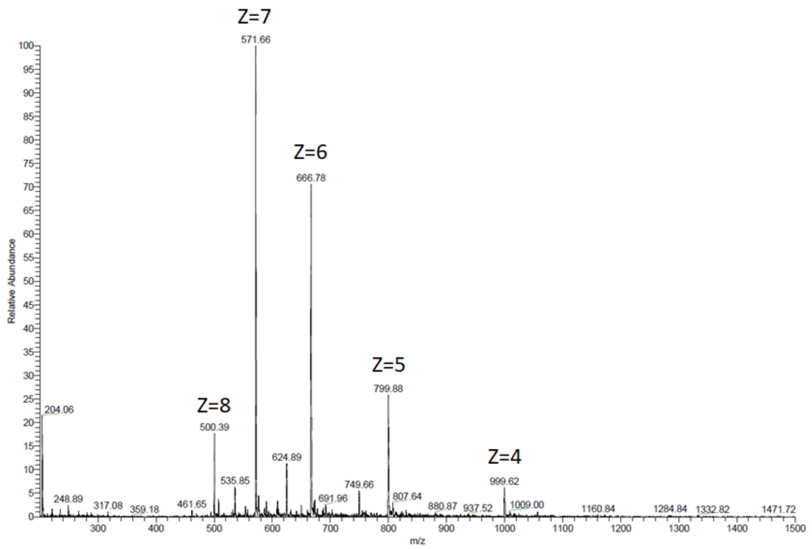


**Fig. S16:** ESI-MS of short competitor 13 mer PNA. $M_{calc}$ =3993.79, $M_{obs}$ =3994.7.

<u>Fluorescence readouts for K13 FIT-PNA 1 and K13 cpFIT-PNA 1 with all 4 competitor PNAs and full-length WT-DNA</u>

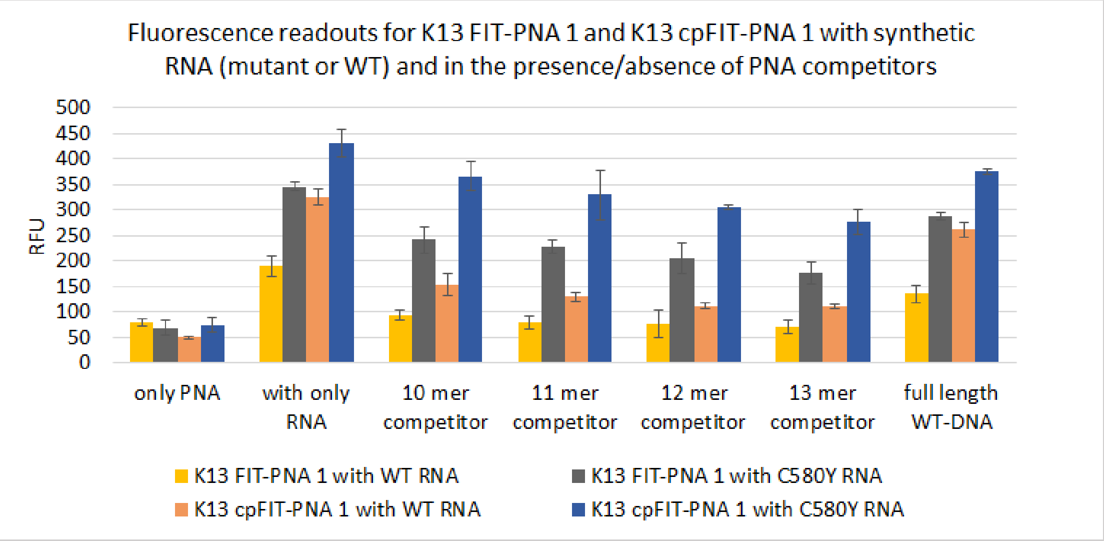


**Fig. S17:** Fluorescence measurements for K13 FIT-PNA 1 and K13 cpFIT-PNA 1 with synthetic RNAs in the presence of competitor PNAs and full-length DNA (17-mer WT sequence). K13 FIT-PNA 1 and K13 cpFIT-PNA 1 on streptavidin plate were annealed to WT or mutant synthetic RNAs in the presence or absence of competitor PNAs (10, 11, 12, or 13 mers).

0.5 µM of each K13 FIT-PNA was incubated at RT for 1 hr for streptavidin binding and washed with PBS (X3). Next, 0.25 µM WT-RNA or C580Y-RNA were added with and without 0.625 µM of the PNA competitors and were left for 2 hr at 37°C for annealing. Fluorescence was measured on a Cytation 3 plate reader, n=3; Ex=587nm, Em=619nm.

Fluorescence readouts for K13 FIT-PNA 2 and K13 cpFIT-PNA 2 with all 4 competitor PNAs

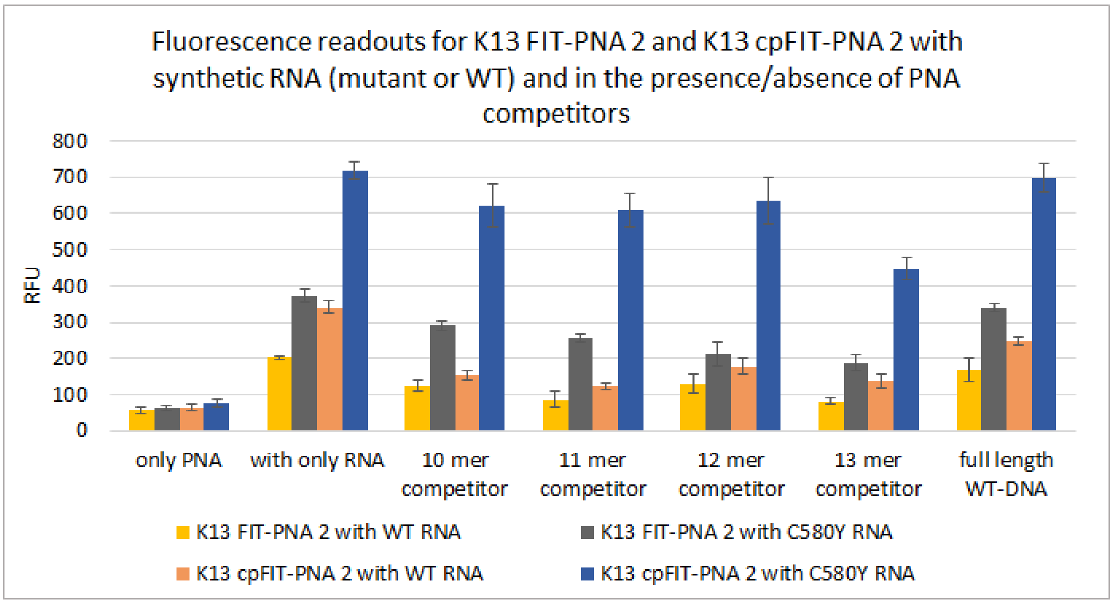


**Fig. S18:** Fluorescence measurements for K13 FIT-PNA 2 and K13 cpFIT-PNA 2 with synthetic RNAs in the presence of competitor PNAs and full-length DNA (17-mer WT sequence). K13 FIT-PNA 2 and K13 cpFIT-PNA 2 on streptavidin plate were annealed to WT or mutant synthetic RNAs in the presence or absence of competitor PNAs (10, 11, 12, or 13 mers).

0.5 µM of each K13 FIT-PNA was incubated at RT for 1 hr for streptavidin binding and washed with PBS (X3). Next, 0.25 µM WT-RNA or C580Y-RNA were added with and without 0.625 µM of the competitors and were left for 2 hr at 37°C for annealing. Fluorescence was measured on a Cytation 3 plate reader, n=3; Ex=587nm, Em=619nm.

**References:**

1. Zheng, H.; M. Saha; D. H. Appella, *Synthesis of Fmoc-Protected (S,S)-trans-Cyclopentane Diamine Monomers Enables the Preparation and Study of Conformationally Restricted Peptide Nucleic Acids.* Org. Lett., 2018. **20**(23): p. 7637-7640.
2. Kolevzon, N.; Hashoul D.; Naik, S.; Rubinstein, A.; Yavin, E., *Single point mutation detection in living cancer cells by far-red emitting PNA–FIT probes.* Chem. Commun., 2016. **52**(11): p. 2405-2407.
3. Tepper, O.; Zheng, H. C.; Appella; D. H.; Yavin, E., *Cyclopentane FIT-PNAs: bright RNA sensors.* Chem. Commun. 2021. **57**(4): p. 540-543; 2023, 59, 11593-11593.
